# Chemical reprogramming enhances homology-directed genome editing in zebrafish embryos

**DOI:** 10.1038/s42003-019-0444-0

**Published:** 2019-05-23

**Authors:** Yagiz A. Aksoy, David T. Nguyen, Sharron Chow, Roger S. Chung, Gilles J. Guillemin, Nicholas J. Cole, Daniel Hesselson

**Affiliations:** 1 Diabetes and Metabolism Division, Garvan Institute of Medical Research, Sydney, NSW Australia; 2 Department of Biomedical Sciences, Faculty of Medicine and Health Sciences, Centre for Motor Neuron Disease Research, Macquarie University, Sydney, NSW Australia; 3 St Vincent’s Clinical School, UNSW Sydney, Sydney, NSW Australia

**Keywords:** CRISPR-Cas9 genome editing, Chemical biology

## Abstract

Precise genome editing is limited by the inefficiency of homology-directed repair (HDR) compared to the non-homologous end-joining (NHEJ) of double strand breaks (DSBs). The CRISPR (clustered regularly interspaced short palindromic repeat)/Cas9 system generates precise, locus-specific DSBs that can serve as substrates for HDR. We developed an in vivo visual reporter assay to quantify HDR-mediated events at single-cell resolution in zebrafish and used this system to identify small-molecule modulators that shift the DNA repair equilibrium in favor of HDR. By further optimizing the reaction environment and repair template, we achieved dramatic enhancement of HDR-mediated repair efficiency in zebrafish. Accordingly, under optimized conditions, inhibition of NHEJ with NU7441 enhanced HDR-mediated repair up to 13.4-fold. Importantly, we demonstrate that the increase in somatic HDR events correlates directly with germline transmission, permitting the efficient recovery of large seamlessly integrated DNA fragments in zebrafish.

## Abstract (web summary)

Aksoy et al. develop a reporter assay that allows them to quantify events mediated by homology-directed repair (HDR) in live zebrafish at single cell resolution. Using this assay, they systematically test and identify small molecule modulators that enhance the efficiency of HDR-mediated genome editing in zebrafish.

## Introduction

Genome-editing tools such as zinc-finger nucleases (ZFNs), transcription activator-like nucleases (TALENs) and clustered regularly interspaced short palindromic repeats (CRISPRs) are powerful technologies that enable targeted gene knockout and precise editing of the genome. While gene knockout can typically be accomplished with high efficiency, precise gene editing remains challenging in many species including the widely used zebrafish model system, where attempts to achieve precise genome-editing often produce a mosaic of precisely and imprecisely edited cells. Efficient genome editing would allow functional validation of human disease-associated variants in animals. Zebrafish are an important model for forward and reverse-genetic analyses of human diseases. However, the inefficiency of precise genome editing has limited its widespread adoption in zebrafish embryos. Similar inefficiency was reported in the initial rabbit, mouse, and human cell culture studies^1–3^.

Most genome editing approaches rely on the generation of a double strand break (DSB) that engages the major DNA repair mechanisms^4,5^; non-homologous end-joining (NHEJ) and homology-directed repair (HDR). The former mechanism typically generates loss-of-function alleles via the incorporation of random indels causing frameshifts leading to the introduction of a premature stop codon at or near the target site, whereas the latter mechanism can be exploited for precise gene editing (insertions, deletions, or point mutations) with an appropriate template^6,7^. NHEJ is the dominant pathway in most organisms, including mouse^2^, zebrafish, vertebrate cells^8^, plants^9^, and insects^10^. An exception is the budding yeast where HDR is the dominant pathway^11^. Furthermore, HDR efficiency varies between cell types. For example, HDR efficiency is higher in mouse embryonic stem cells as compared to primary mouse cells^8^. Importantly, the equilibrium between these pathways is genetically regulated, as the selective knockdown of genes associated with NHEJ (e.g., Lig4 or Ku) shifts the balance in favor of the HDR pathway in insects^10^ and plants^12^. Small-molecule modulation of validated NHEJ/HDR regulators is emerging as a more flexible experimental approach for enhancing HDR efficiency. SCR7, a chemical inhibitor of Lig4, improved HDR efficiency in mouse embryos^2^, although conflicting effects are observed in other cell types^1,13–15^, suggesting that SCR7 exerts species-specific effects. Similarly, the DNA-dependent protein kinase (DNA-PK) inhibitors, KU0060648^16^ and NU7441^17^, which also block NHEJ, increase the efficiency of Cas9-mediated HDR in HEK293T cells and mouse embryonic fibroblasts^18^. Complementary attempts to directly stimulate HDR by activating RAD51 were also successful in certain contexts^1,19^.

The zebrafish is a powerful system to model human disease^20^. In addition to high physiological and anatomical conservation with humans, zebrafish have a fully sequenced genome in which 82% of human disease genes have clear homologs^21^. Their small size, ease of breeding, and high number of offspring makes zebrafish ideal for high-content, rapid drug screening. The vast genetic toolbox available for the manipulation of zebrafish allow forward- and reverse-genetic screen studies, while its transparent embryos make zebrafish an excellent organism for in vivo imaging^20^. Recent advances in genome editing technologies have opened a new avenue to study gene function in zebrafish. Since ZFN/TALEN/CRISPR technologies have been established, more than one hundred zebrafish genes have been knocked out using the NHEJ pathway^22^. However, there are limited reports of HDR-driven insertions or point mutations, which were generated using low-efficiency approaches^23–28^.

Here we establish a quantitative system for assessing HDR efficiency in live zebrafish embryos at single-cell resolution. We use this system to optimize conditions for CRISPR-mediated HDR by chemically reprogramming the zebrafish embryo to favor template-directed genome editing. Through this approach, we report the optimization of a protocol for efficient editing of the zebrafish genome.

## Results

### A system for quantitative in vivo analysis of HDR

**Fig. 1:**
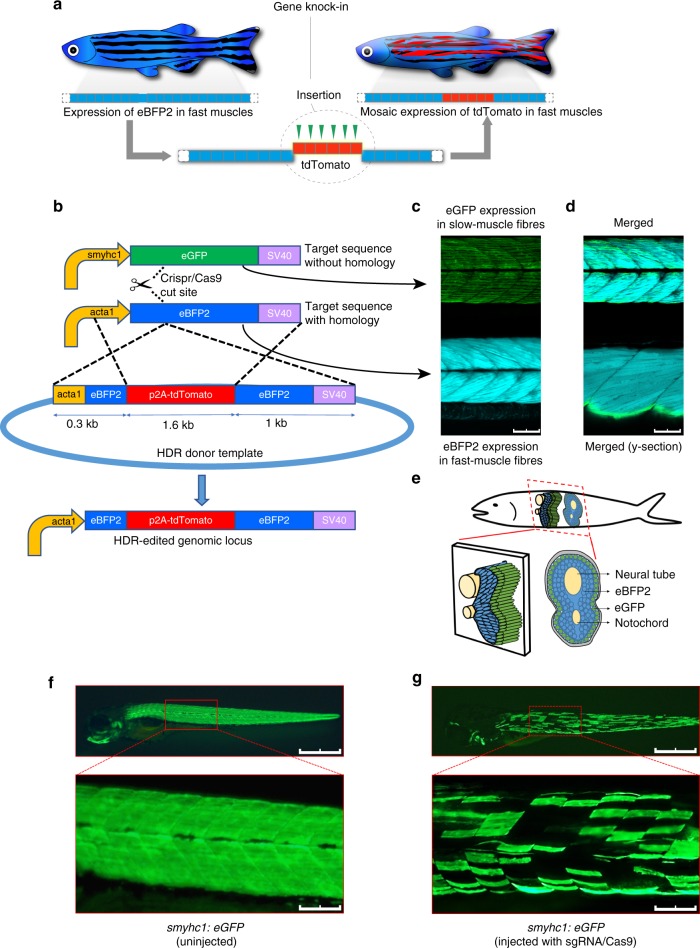
Overview of the in vivo homology-directed repair (HDR) detection system. **a** Schematic representation of the visual HDR readout. **b** The single guide RNA (sgRNA)-Cas9 complex targets the identical sequence in *eGFP* and *eBFP2*. Embryos are co-injected with HDR donor template to integrate the tdTomato transgene into the *acta1-eBFP2* locus. **c** Confocal sections showing *smyhc1:eGFP* and *acta1-eBFP2* expression and **d** merged images. Scale bars: 75 µm. **e** Cross-sectional representation of a zebrafish embryo showing slow and fast muscles. **f** Expression of *smyhc1-eGFP* in slow-muscle fibers (3 dpf). **g** Mosaic loss of *smyhc1-eGFP* expression in slow-muscle fibers (3 dpf) of embryos injected with a CRISPR (clustered regularly interspaced short palindromic repeat)/Cas9 complex targeting *eGFP*. Scale bars: 500 µm, main image and 100 µm, inset

To establish a simple and quantitative readout for HDR, we focused on the fast-muscle fibers in the zebrafish trunk. We devised a gene replacement strategy whereby eBFP2 expressed specifically in the fast-muscle fibers of a stable transgenic zebrafish is disrupted by the insertion of tdTomato, converting the muscle fiber from blue to red fluorescence (Fig. 1a). The donor template incorporated a CRISPR/Cas9 target site at the tdTomato insertion site in the homology arm that was shared with the *eBFP2* genomic target locus (Fig. 1b) since this approach was reported to increase HDR efficiency^24^. When a single guide RNA (sgRNA) targeting *eBFP2* was co-injected with Cas9 protein, CRISPR/Cas9 linearized donor DNA serves as a template for HDR-mediated gene editing (Fig. 1b). To test the feasibility of this approach, we used a double transgenic zebrafish strain that expressed eBFP2 under the control of the fast-muscle *acta1* promoter^29^ and eGFP under the control of the slow-muscle *smyhc1* promoter^30^ (Fig. 1c, d). Zebrafish slow muscle is a single layer of parallel fibers that encase the fish beneath the skin, rendering them accessible to rapid and accurate quantitation by fluorescence microscopy (Fig. 1e). To evaluate the efficiency of the sgRNA, we targeted a region that is identical between *eBFP2* and *eGFP* (Fig. 1b) and confirmed the loss of eGFP fluorescence in a mosaic pattern across individual slow-muscle cells at 72 h post fertilization (hpf) (Fig. 1f, g). Similar loss of eBFP2 expression in fast-muscle cells was observed (Supplementary Movie 1). Next, we tested whether a donor DNA fragment encoding the red fluorescent protein tdTomato flanked by a 303 bp left homology arm (LHA) and 1022 bp right homology arm (RHA) could be inserted into the *acta1:eBFP2* transgene (Fig. 1b). We observed red fluorescent signal in individual fast-muscle fibers that showed loss of eBFP2 expression (Supplementary Movie 2), demonstrating successful insertion of the tdTomato transgene into the *eBFP2* locus (and therefore causing loss in expression of eBFP2). Control injections without *eBFP2* sgRNA did not generate any red fluorescent signal (Supplementary Movie 3). Our data are consistent with integration of the tdTomato transgene into a CRISPR/Cas9-dependent genomic lesion at low efficiency (4.0 ± 3.0 red fibers per embryo). Importantly, this assay provides rapid quantification of in vivo genomic editing at single-cell resolution.

### Small-molecule enhancement of HDR efficiency

**Fig. 2:**
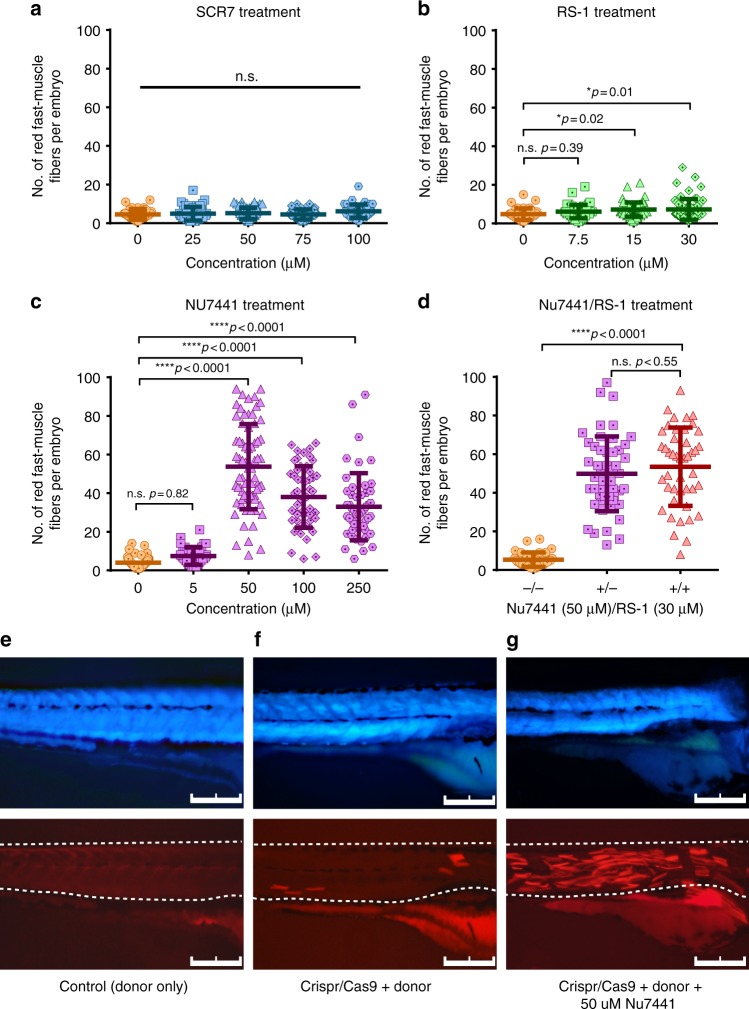
Chemical enhancement of CRISPR (clustered regularly interspaced short palindromic repeat)/Cas9-mediated homology-directed repair (HDR) efficiency. Effects of **a** SCR7, **b** NU7441, **c** RS-1 on the efficiency of CRISPR/Cas9-mediated tdTomato expression in zebrafish embryos. **d** Co-injection of optimized concentrations of NU7441 and RS-1 in zebrafish embryos. Error bars represent standard error of the mean. **e**–**g** Fluorescent microscopy of zebrafish embryos injected with **e** tdTomato donor template, **f** CRISPR/Cas9 with tdTomato donor template, or **g** CRISPR/Cas9 with tdTomato donor template and optimized concentration of NU7441 at 50 µM. Scale bars: 250 µm

Inhibition of NHEJ and stimulation of HDR by small molecules appears to have context-specific outcomes for precise genome editing^1,2,13–15^. We used the fast-muscle fiber assay described above as a test bed to identify the optimal small-molecule treatment for enhancing HDR in zebrafish. Transgenic *acta1:eBFP2* embryos were co-injected with SCR7, RS-1, or NU7441 (Fig. 2a–c), at a range of doses up to the solubility limit of each drug. None of the treatments affected embryo survival (Supplementary Fig. 1). Furthermore, injection of donor DNA alone did not generate any red fibers (Fig. 2e), highlighting the specificity of the assay. Therefore, we proceeded to quantify the total number of muscle fibers expressing tdTomato in the trunk of each embryo (*n* ≥ 40 zebrafish per condition; Fig. 2f). SCR7 administration had no effect on red fiber number compared to the dimethyl sulfoxide (DMSO) vehicle control (Fig. 2a). At the highest tested doses, RS-1 showed a modest but statistically significant increase in the number of red fibers (Fig. 2b; DMSO, 4.8 ± 3.0; 15 µM, 7.2 ± 3.7, *p* = 0.02; 30 µM, 7.3 ± 5.3, *p* = 0.01). In contrast, NU7441 administration showed a dramatic increase (Fig. 2f, g), with the maximal effect at 50 µM (Fig. 2c, DMSO, 4.0 ± 3.0; 50 µM, 53.7 ± 22.1, *p* < 0.0001). We did not observe a further increase in the number of red fibers by adding RS-1 to the optimal NU7441 dose (Fig. 2d).

### Qualitative analysis masks the effects of HDR stimulation

Highlighting the importance of quantitative single fiber analysis, qualitative analysis (presence vs. absence of red fibers) did not reveal an HDR-stimulating effect for any of the drug treatments (Supplementary Fig. 1). For example, a comparison of qualitative assessment of DMSO control group without drugs, RS-1 at 30 µM and NU7441 at 50 µM display similar rates of red fiber induction (Supplementary Fig. 1; DMSO, 69.5%, *n* = 130; RS-1 30 µM, 75.5%, *n* = 352; Nu 50 µM, 80.0%, *n* = 314). However, amongst the animals that exhibited red fluorescence, we observed a difference in the number of red fibers between the control and drug-treated embryos that was consistent with the single fiber analysis. For example, the DMSO control embryos that exhibited red fast-muscle fibers (69% of total) had an average of 4.0 ± 3.0 fibers per embryo, whereas NU7441 (50 µM) administered embryos that exhibited red fast-muscle fibers (80% of total) had an average of 53.7 ± 22.1 fibers per embryo. We conclude that qualitative assays reduce the dynamic range of whole-embryo assays and can mask the effects HDR-stimulating conditions.

### Zebrafish-specific optimizations for Cas9-mediated HDR

To optimize Cas9-mediated HDR efficiency in zebrafish, we first compared circular and linear plasmid DNA donors. We found no difference between donor type in DMSO controls (Supplementary Fig. 2B; linear, 4.2 ± 0.7, *n* = 25; circular, 4.9 ± 0.5, *n* = 34). However, co-injection with circular plasmid DNA yielded a significantly higher number of red fast-muscle fibers than linearized plasmid DNA in NU7441 (50 µM)-administered embryos (Supplementary Fig. 2B; linear, 39.0 ± 3.3, *n* = 30; circular, 51.0 ± 3.2, *n* = 43, *p* < 0.001). Our results also indicated that linearized plasmid DNA show more toxicity compared to circular plasmid DNA in all experimental groups (Supplementary Fig. 2B). It is important to note that overall HDR efficiency is relative to the cutting efficiency of CRISPR/Cas9, which is highly variable across different loci. In this experiment, we tested six different sgRNA targeting *eBFP2* (Supplementary Table 1) and used the sgRNA showing the highest efficiency (sg_eBFP2_04).

The rapid embryonic development of zebrafish poses a challenge for genome editing. The post-fertilization cell cycle length is less than an hour in fertilized zebrafish embryos^31^ compared to 16–20 h in mice^32^. This rapid proliferation provides a 20 min experimental window in which to inject DNA into single-cell eggs (~20–40 min post fertilization), and therefore it is conceivable that variability in the timing of the DNA injection within the cell division cycle in this short time period could contribute to mosaicism and low germline transmission rates in zebrafish. We tested whether slowing the first cell division of zebrafish embryos, by incubation on ice, enhanced Cas9-mediated HDR efficiency by allowing more time for the formation CRISPR/Cas9 assembly followed by DNA repair by HDR. Our results showed ice incubation for 15–20 min significantly enhances HDR efficiency in NU7441 and NU7441/RS-1 administered embryos with only minimal impact on development and survival (Supplementary Fig. 3A–B; NU7441 50 µM RT, 36.6 ± 2.6, *n* = 54; NU7441 50 µM ice, 50.1 ± 3.6, *n* = 43, *p* = 0.0019). These results showed Cas9-mediated HDR can be increased 1.5-fold to 2-fold by slowing embryo development temporarily. Therefore, we conducted all further experiments using the low-temperature injection protocol.

### Phenotypic and molecular analysis of integration events

**Fig. 3:**
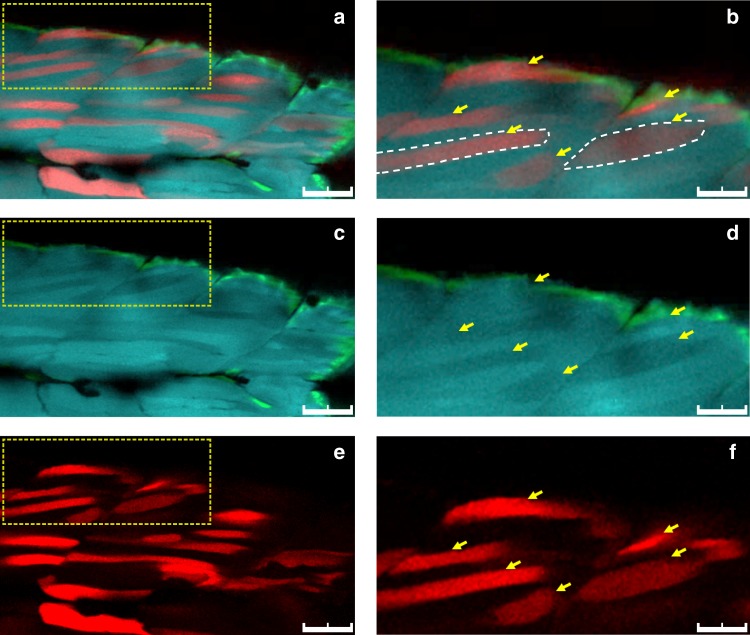
Single fiber analysis of homology-directed repair (HDR) events. Cross-sectional in vivo imaging of a zebrafish embryo (3 dpf) co-injected with NU7441 at 50 µM and RS-1 at 30 µM. **a** Merged image showing mutually exclusive expression of eBFP2 and tdTomato in individual fast-muscle fibers (yellow arrows). **c**, **e** Individual channels showing eBFP2 expression (**c**) and tdTomato (**e**). Adjacent slow-muscle fibers express eGFP. **b**, **d**, **f** Higher magnification confocal sections of regions indicated in **a**, **c**, **e**. Scale bars: 50 µm

The transmission of the *acta1:eBFP2* transgene is consistent with a single-copy Tol2-mediated insertion in the zebrafish genome^29^. Therefore, donor integration should eliminate eBFP2 expression in tdTomato-expressing cells. As predicted for HDR-mediated integration, in NU7441-injected embryos we observed a mutually exclusive pattern of red and blue fluorescence in individual fast-muscle fibers (Fig. 3a–f). Furthermore, the homology arms were essential for targeting because no integration into the slow-muscle *smyhc:eGFP* transgene, which contained an identical CRISPR/Cas9 target site, was observed (Fig. 3a–f). To further confirm that tdTomato integration requires homology with the target site, we used a validated sgRNA targeting the endogenous zebrafish *golden* locus^26^, which is essential for pigmentation. We did not observe any red fast-muscle fibers despite disrupting pigmentation (Supplementary Fig. 4A–C). Therefore, we conclude that tdTomato is not efficiently integrated into non-homologous double-stranded breaks.

**Fig. 4:**
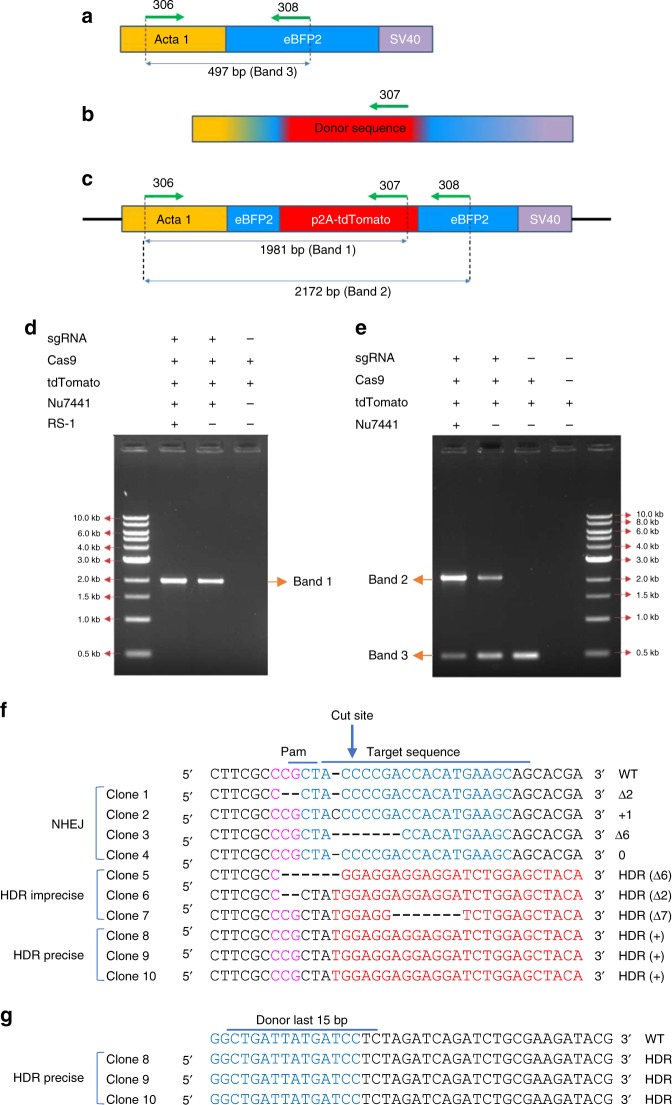
Molecular analysis of homology-directed repair (HDR)-mediated integration events. **a**–**c** Primer design for PCR amplicons and sequencing analysis of HDR events. DNA fragments from **a** the *acta1:eBFP2* transgene, **b** the tdTomato donor template, and **c** the predicted CRISPR (clustered regularly interspaced short palindromic repeat)/Cas9-mediated HDR integration of tdTomato are depicted showing the positions of primers 306, 307, and 308. **d** PCR analysis using primers 306/307. The primer 306 binding site is not present in the donor sequence, whereas the primer 307 binding is unique to the donor template. Thus, PCR amplification of a 1981bp band is only expected in embryos with targeted integration events. **e** PCR analysis using primers 306/308. Primer 308 binds to an *eBFP2* sequence that is present in both the *acta1:eBFP2* transgene and the tdTomato donor. Therefore, the 306/308 primer pair can generate two products: a 497 bp amplicon (no template integration) and a 2172 bp amplicon (HDR-mediated integration of the tdTomato transgene). **f** Sequence alignment of the 5′ end of genome-edited clones. (Magenta) Pam site, (blue) sgRNA binding site, and (red) integrated donor fragment. **g** Sequence alignment of the 3′ end of clones that showed precise HDR in **f**

Next, we designed PCR-based assays to investigate whether blue to red color switching in fast-muscle fibers is mediated by precise HDR (Fig. 4a–c). Primers 306 and 307 generate a 1981 bp PCR amplicon (Band 1), which is only amplified when the donor-derived tdTomato coding sequence is inserted downstream of the transgenic *acta1:eBFP2* promoter (Fig. 4c). We observed a band of the expected size (Band 1) in embryos with red fast-muscle fibers, which was not observed in controls lacking the *eBFP2*-targeting sgRNA (Fig. 4d), indicating that amplifcation requires genomic integration. To investigate whether the number of red fast-muscle fibers corresponds to the proportion of edited alleles, we used a second set of PCR primers (306 and 308) to simultaneously amplify the original (Band 3, 497 bp) and edited (Band 2, 2172 bp) trangenic *acta1* alleles (Fig. 4a–c). NU7441 increased the intensity of the edited Band 2 amplicon compared to controls (Fig. 4e). Quantification of individual embryos revealed a 7.7-fold increase in the intensity of Band 2 amplicon in NU7441-injected embryos (Supplementary Fig. 5), which was similar to the effect on red fast-muscle fiber number (9.3-fold, Fig. 2c).

### Enhanced germline transmission of precise HDR-edited alleles

To determine whether the edited alleles were generated by precise HDR, we sequenced both ends of the DSB from tdTomato-positive embryos. Three out of 10 edited alleles showed seamless integration of transgene (Fig. 4f, g). This is consistent with our results from scanning 3D-rendered y and z stacks of embryos for precise replacement of blue fluorescent signal with red fluorescence in fast-muscle fibers. (Supplementary Movies 4 and 5).

Finally, to examine whether transient chemical reprogramming increased the germline transmission rate of HDR-edited alleles, we raised injected embryos to adulthood and screened their progeny for the expression of tdTomato in fast-muscle fibers. Germline transmission was analyzed in outcrosses with wild-type (non-transgenic) breeding partners (Supplementary Table 2). In the DMSO control group, only 1/16 fish produced tdTomato-expressing embryos (42/120 embryos from the single germline-transmitting fish), whereas 3/6 fish in the NU7441 group produced tdTomato-expressing embryos (19/24, 81/110, and 68/75 embryos from the three germline-transmitting fish: *χ*^2^ *p* = 0.018 compared to DMSO control). As predicted from the single fiber analysis, the addition of RS-1 did not increase the germline transmission rate as compared to NU7441 alone (4/10 fish produced 61.5% (*n* = 200) tdTomato-expressing embryos; *χ*^2^ *p* = 0.034 compared to DMSO control).

## Discussion

The low efficiency of HDR-mediated events is a major barrier to the functional analysis of human disease-associated variation in model systems. Here, we address this challenge in zebrafish. We first developed a robust assay that simultaneously provides qualitative and quantitative readouts in vivo to evaluate potential HDR-stimulating interventions. We tested several small molecules that have been reported to modulate DNA repair pathways by direct injection into fertilized zebrafish embryos. We obtained improved CRISPR-mediated gene-editing with up to 13.4-fold enhancement with NU7441, an NHEJ inhibitor. Other DNA repair modulators, such as the NHEJ inhibitor SCR7, showed minimal or no effect on HDR efficiency. One possible explanation for this discrepancy is that NU7441 and SCR7 have different targets in the NHEJ pathway. NU7441 inhibits the DNA protein kinase catalytic subunit (DNA-PKcs), which appears earlier in the NHEJ repair pathway than the ligation complex that SCR7 targets for inhibition. Alternatively, the mode of delivery, treatment duration, or species-specific differences in target binding affinities may contribute to the observed context-specific effects of SCR7. Previous studies have shown that inhibition of DNA ligase 4 by SCR7 can enhance HDR-mediated genome editing in HEK293 cells^13,18^, mice^2^, and recently in zebrafish^27^. Nevertheless, recent studies also reported that SCR7 has limited or no effect CRISPR/Cas9-mediated HDR events in HEK293A cells^19^, HEK293T cells^14^, neural progenitor cells and human pluripotent stem cells^33^ CHO cells^15^, and rabbits^1^. RS-1 also provides a modest increase in HDR in U2OS and HEK293 cells^19^, rabbit embryos^1^, and zebrafish embryos^27^. Our results are concordant with these studies in that RS-1 enhanced Cas9-mediated HDR 1.5-fold in zebrafish embryos. NU7441 has only been tested in HEK293/17 and MEF cells, which resulted up to 4-fold enhancement of HDR^18^. The effect of NU7441 on animal models was hitherto unknown.

To the best of our knowledge, we report the first application of NU7441 as a potent HDR enhancer in zebrafish. Our results showed that NU7441 can improve Cas9-mediated HDR up to 13.4-fold in zebrafish, which is ~9 times more effective than RS-1 alone. Furthermore, combined treatment with both NU7441 and RS-1 did not further enhance HDR as compared to NU7441 alone (Fig. 2). This suggests that inhibition of NHEJ is more effective than enhancing HR in order to increase HDR-driven knock-in events. The methods used for analyzing HDR efficiency varies across previous reports and likely contributes to variable outcomes reported by different groups. Therefore, the effect of small molecules on HDR efficiency should be evaluated within the context of the model/cell type used.

We further observed that results from our quantitative model are consistent with the downstream germline transmission rates. Drug treatments that included NU7441 increased germline transmission rates, although the data are limited by the small numbers of injected animals that were raised to adulthood. Therefore, generalizing this conclusion will require larger sample sizes and the evaluation of additional loci. Qualitative assessment of HDR efficiency could not detect the treatment effects or predict the germline transmission rates. This may explain the previously reported low levels of germline transmission of HDR-edited alleles in zebrafish. Thus, qualitative assessment of knock-in efficiency alone, while being visual and informative, is not sufficient to accurately determine the actual HDR-mediated integration events.

In addition to chemical modulation of the intracellular environment, we addressed a physical constraint that limited genome editing in zebrafish: the short cell cycle. We hypothesized that slowing down the cell cycle by reducing embryo temperature would reduce mosaicism by increasing the likelihood that desired HDR events would affect the majority of daughter cells. We identified incubation parameters that caused minimal mortality while increasing the number of HDR-mediated events in our quantitative assay. For seamless integration of large constructs into the target locus, we used long homology arms with different lengths as this approach was previously suggested to increase integration events in zebrafish^24^. Although there have been conflicting reports on the relative efficiencies of linearized and circular DNA in zebrafish^24,26,27^, our quantitative single fiber analysis clearly showed that circular DNA improves integration efficiency. Together, the chemical and environmental optimizations presented here provide proof-of-concept for the seamless integration of large (>1.5 kb) constructs into the zebrafish genome.

The seamless and precise integration of large DNA fragments will fill a critical gap in the zebrafish toolbox, allowing the tagging of endogenous zebrafish genes with exogenous constructs, such as fluorescent reporters, light-responsive proteins (for optogenetic research), or other DNA elements that are driven by native zebrafish promoters. In conclusion, we have developed a visual reporter system allowing rapid and easy quantitative analysis of knock-in efficiency at single-cell resolution in vivo. We identified small molecules to change the DNA repair equilibrium in favor of HDR pathway and determined the optimum conditions to dramatically enhance HDR-mediated knock-in efficiency in zebrafish. Taken together, our quantitative system can be used as a rapid and informative reader of the desired outcome and act as a test bed for rapid and high-throughput screening of many drug candidates to further improve HDR, while our optimized HDR-enhancing strategy will enable seamless integration of precise genetic modifications at endogenous loci.

## Methods

### Zebrafish maintenance

Zebrafish embryos and adults were maintained and handled under standard conditions according to protocols approved by Animal Research Ethics Committee at Macquarie University (ARA: 2015-034) and in compliance with the Animal Research Act, 1985 and the Animal Research Regulation, 2010. The *Acta1:eBFP2;smyhc1:eGFP* line was generated by crossing *Tg(acta1:eBFP2)*^*pc5* ^^29^ and *Tg(smyhc1:eGFP)*^30^.

### Target site selection

CRISPR sgRNA target sites were selected manually within the 5′ region of the *eBFP2* gene that match the sequence GN18GNGG according to Schier et al^34^. To avoid off-target effects, these sites were analyzed by BLASTN (Zv9) using Bowtie and Bowtie2 methods, and the pre-defined specificity rules that do not tolerate any mismatches in the first ten 3′ bases of the target site.

### Production of sgRNA

To generate templates for sgRNA transcription, target gene-specific complementary oligonucleotides containing the 20-base target site without the PAM (Supplementary Table 3) were annealed to each other, and then cloned into a plasmid containing T7 promoter sequence and tracrRNA tail. The resulting sgRNA template was purified using Zyppy Plasmid MiniPrep Kit (Zymo Research). The size and quality of resulting sgRNA was confirmed by electrophoresis through a 3% (wt/vol) low-range agarose gel. Recombinant Cas9 protein was obtained from Toolgen Inc.

### Construction of donor vector

The donor vector was ordered from GeneArt. For the *eBFP2* locus, the donor vector was designed to have a reporter construct encoding tdTomato fused with the viral 2 A peptide, which would be inserted in the CRISPR cut site, flanked by 0.3 kb LHA and 1.0 kb RHA of *eBFP2* DNA fragments. Our design was influenced by Shin et al. ^24^, incorporating homology arms with unequal sizes while limiting the total size to 3 kb.

### Microinjection

On the day of injections, the injection mix was prepared as described in Table 1. For the initial screen, *Acta1:eBFP2;smyhc1:eGFP* embryos were collected. Injection components were mixed and incubated at room temperature for 5 min to form complex, and then stored on ice for ~15 min. The injection mix was loaded into the needle and microinjected into zygotes using standard zebrafish injection protocols established in our laboratory. Delivery of 1 nl of injection mixture into the single cell (not the yolk) was performed and the injected eggs were grown in 1× egg water in 100 mm plastic Petri dish and kept in the incubator at 28 °C. Embryo density did not exceed more than 60 embryos in 25 ml egg water per petri dish. Uninjected embryos (control group) were kept from the same clutch and grown at 28 °C as controls. Embryos were grown to 24–72 hpf. Embryos with developmental defects were removed after at the end of 24, 48, and 72 hpf. At 72 hpf, embryos were randomly selected and anesthetized using Tricane.

**Table 1:** Contents of the embryo injection mastermix used

Contents	Knockout mix		Knock-in mix		Knock-in mix with drugs	
	1× (µl)	[Final] (ng/µl)	1× (µl)	[Final] (ng/µl)	1× (µl)	[Final] (ng/µl)
CRISPR sgRNA	3.5	75	3.5	75	3.5	75
Cas9 protein	1	500	1	500	1	500
Donor	–	–	1	25	1	25
Drugs	–	–	–	–	1	Desired range
Phenol Red	1.5	n/a	0.5	n/a	0.5	n/a
Water	3	n/a	3	n/a	2	n/a

*n/a* not available, *CRISPR* clustered regularly interspaced short palindromic repeat

### Genotyping

To identify the somatic mutagenesis in CRISPR/Cas9 injected embryos, genomic DNA (gDNA) was extracted from pools of eight embryos using the HotSHOT method. The resulting gDNA was analyzed using HRM assay. The successful hits from HRM assay are further genotyped by polymerase chain reaction (PCR). For PCR-based genotyping, two different primer combinations (306/308 and 307/308) were used to confirm the precise integration of the donor to the target cut site and to measure the integration rate (mosaicism), respectively. Uncropped gel images are presented in Supplementary Fig. 6.

### Microscopy

Images were taken on a Leica DMi3000 inverted microscope and Zeiss 880 confocal microscope. Fish embryos were embedded on 1% low-melting agarose in 35 mm glass bottom petri dish. Virtual cross-section of the fish embryos were generated and analyzed using the Imaris software.

### Reporting summary

Further information on research design is available in the Nature Research Reporting Summary linked to this article.

## Supplementary information

Supplementary Information

Description of additional supplementary items

Reporting Summary

Supplementary Data 1

Supplementary Movie 1

Supplementary Movie 2

Supplementary Movie 3

Supplementary Movie 4

Supplementary Movie 5

## Data Availability

The data and reagents generated during the current study are available from the corresponding author upon reasonable request. The individual data points plotted in the figures are reported in Supplementary Data 1.
