# Supplementary material for: Chemical reprogramming enhances homology-directed genome editing in zebrafish embryos
Source: Commun Biol. 2019 May 23;2:198. doi: 10.1038/s42003-019-0444-0 (PMC6533270; doi:10.1038/s42003-019-0444-0)
Supplement: Supplementary file 1 — Supplementary Information [file 42003_2019_444_MOESM1_ESM.pdf]

Supplementary Figure 1

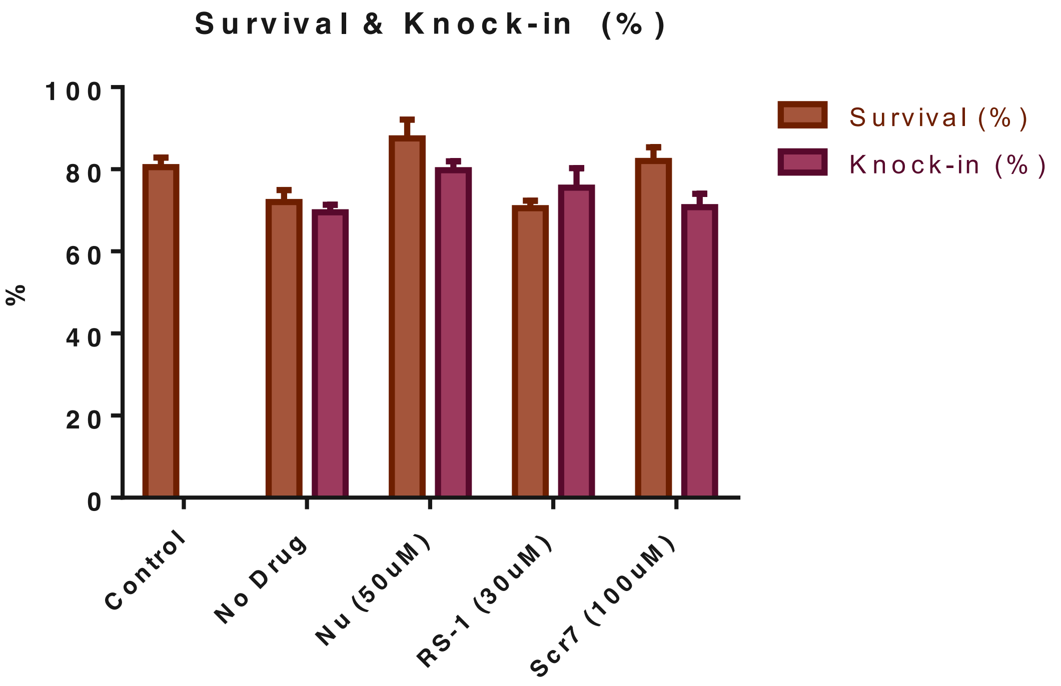

The survival and knock-in rates (%) of zebrafish embryos injected with Nu7441 (50 μM), RS-1 (30 μM) and Scr7 (100 μM) was compared to the control groups. (A) The results were grouped together for survival and knock-in rate per each experimental group and charted on the same graph. The results suggested the drugs do not affect the survival rate. Although Nu7441 (50 μM) injected embryos show a higher survival rate, it is not statistically significant ($p>0.05$). The knock-in rates follow a similar trend except only Nu7441 (50 μM) injected embryos show a significantly higher knock-in rates compared to both control groups whereas the other drugs do not show any significant difference compared to the no-drug control group.

Supplementary Figure 2

Effect of plasmid linearization to knock-in & survival rates

A

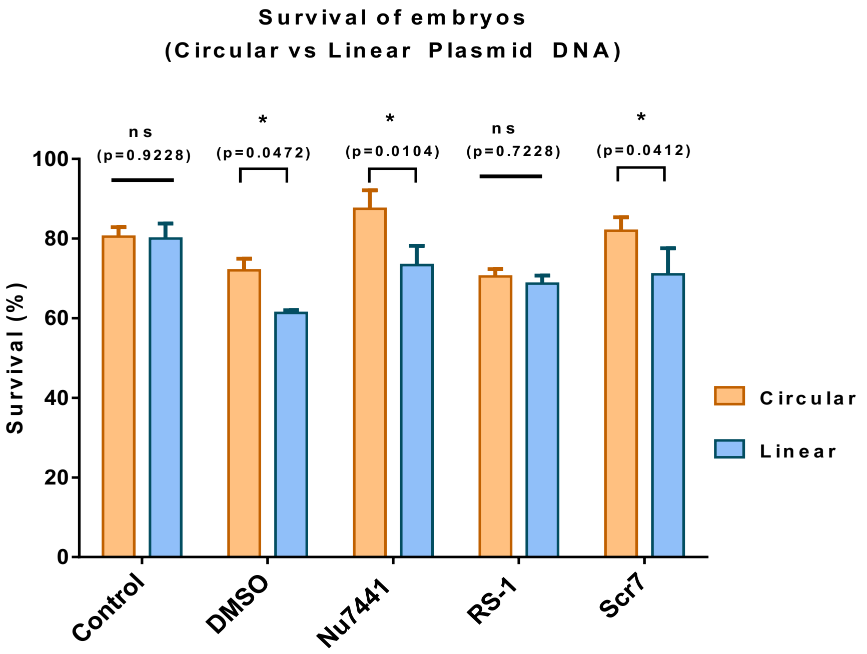

B

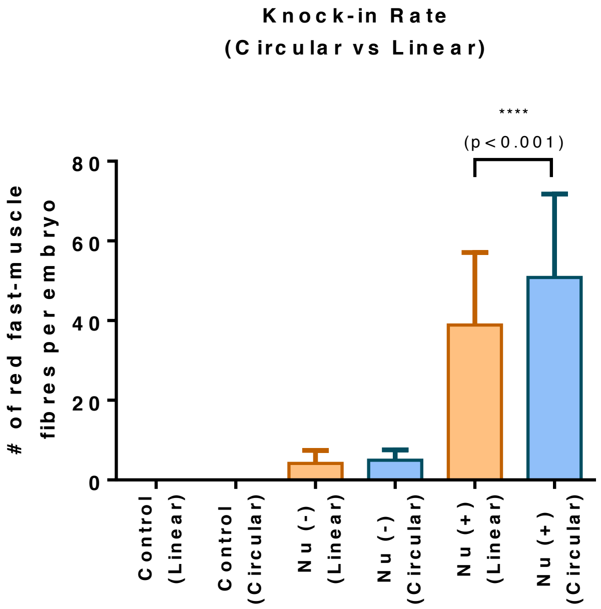

In this experiment, the effect of linearizing the plasmid donor DNA on knock-in rates was investigated. The plasmid donor template was linearized from the left homology arm using Bsu36I enzyme. The linearized and circular plasmids were co-injected with optimum concentrations of Nu7441 (50 µM), RS-1 (30 µM) and Scr7 (100 µM) and DMSO control (without drugs). (A) Circular plasmid donor DNA shows less toxicity in comparison to co-injection with linearized plasmid DNA. (B) Knock-in rate quantified based on the # of red fast-muscle fibers shows a significant difference between circular and linear plasmids, circular plasmids showing higher knock-in rates.

Supplementary Figure 3

Effect of ice incubation on survival over time

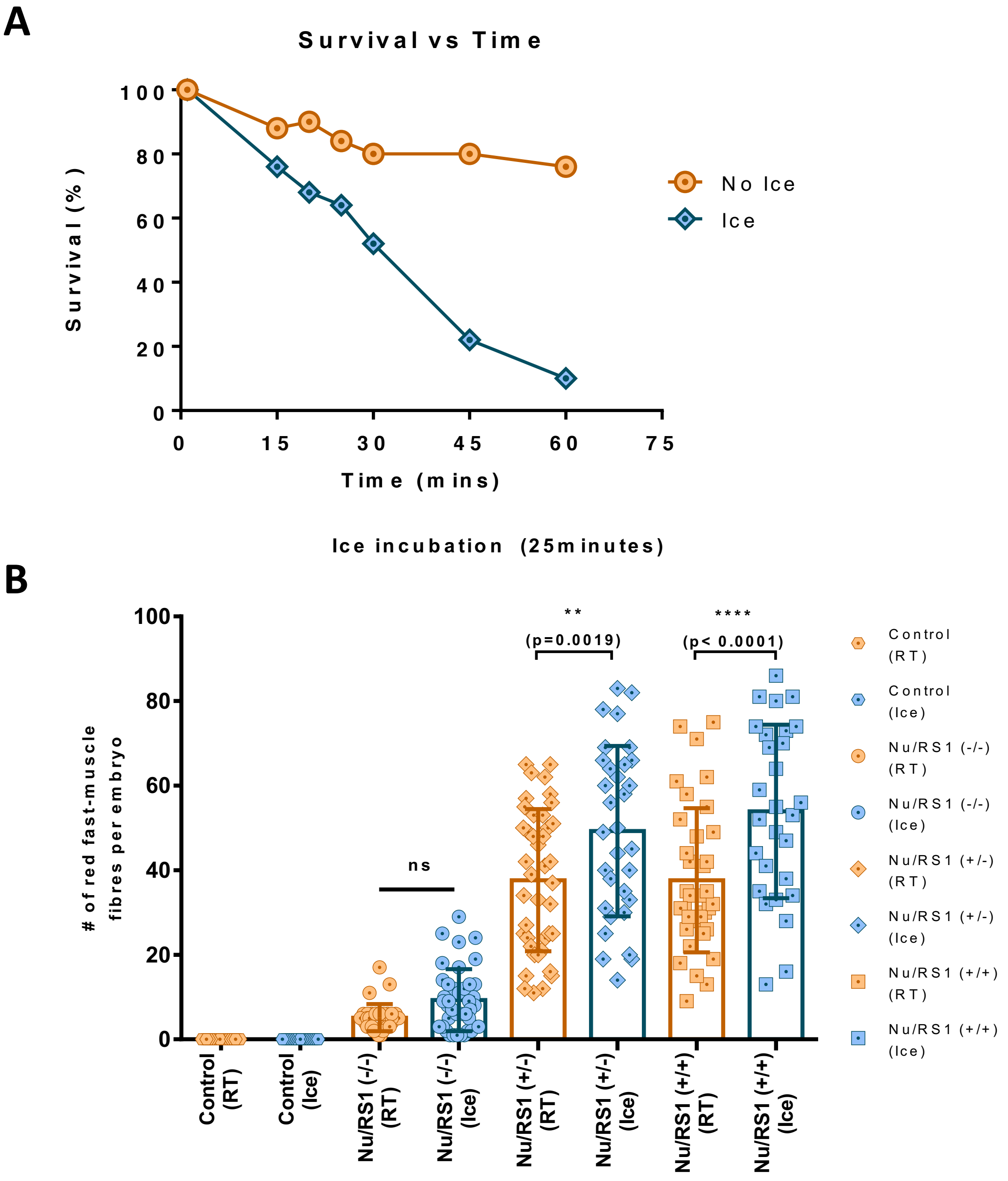

In this experiment, the effect of ice incubation on the survival of zebrafish embryos was measured over time. (A) More than 75% of zebrafish embryos was alive after incubating at RT for 60mins while 90% of the embryos incubated on ice were dead. More than 50% of the embryos can tolerate ice incubation well for the first 30mins. (B) Incubating zebrafish embryos on ice for 25 minutes enhanced Cas9-mediated HDR in Nu7441 and Nu7441 & RS-1 administered embryos up to 2-fold in comparison to embryos injected at room temperature.

Supplementary Figure 4

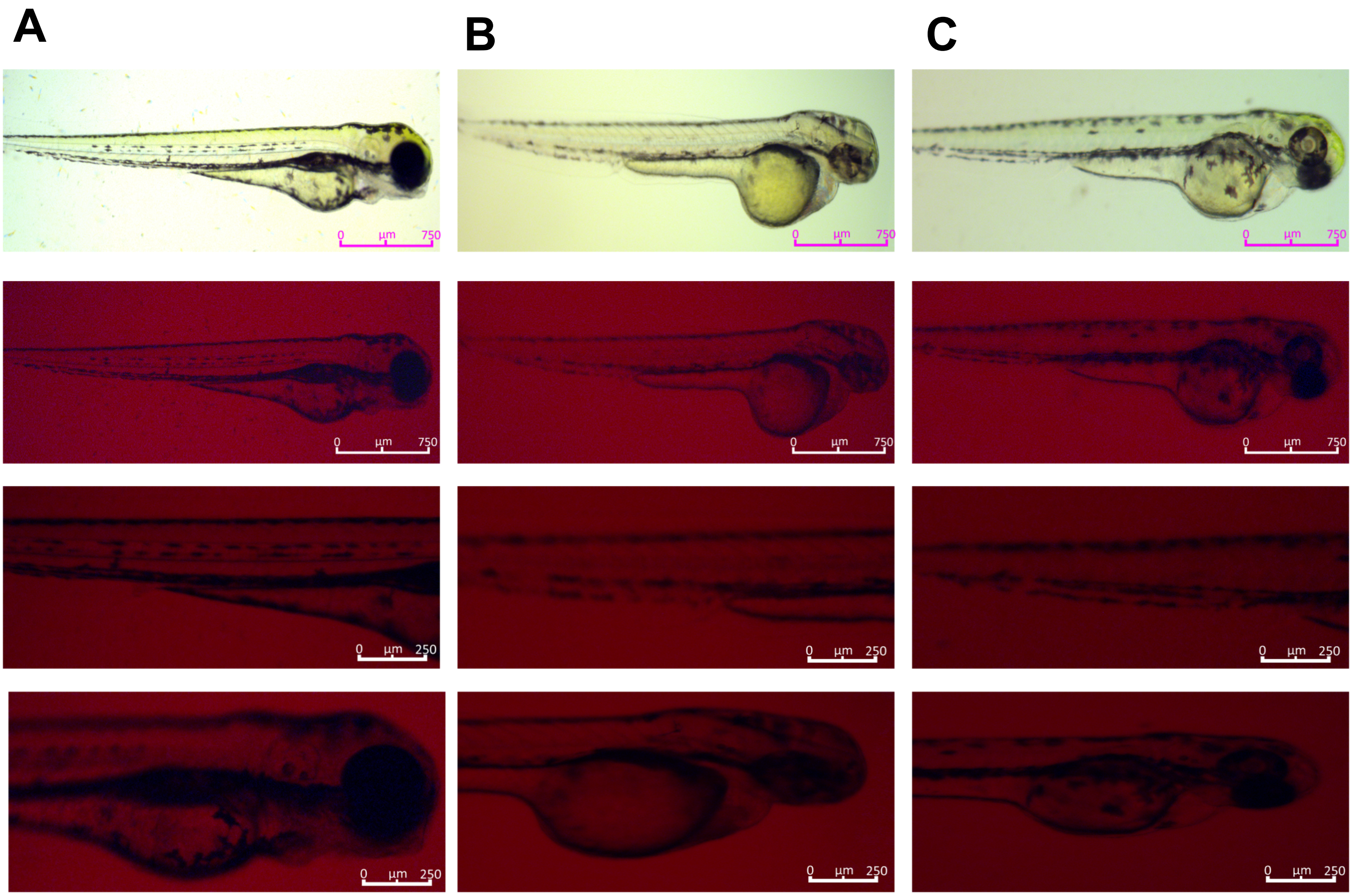

To test if any random donor (tdTomato) insertion is seen when the donor is co-injected with a known Crispr (targeting golden locus) that is not specific to the eBFP2 target site, zebrafish embryos were co-injected with the following material:

1) (A) WT: Tomato
2) (B) WT: Golden Crispr/Ca9 + Tomato
3) (C) WT: Golden Crispr/Ca9 + Tomato + Drugs

Multi-channel analysis of bright-field and red-fluorescence showed no red fibers in any of the experimental groups. Scale bars: 750 μm, main image and 250 μm, inset.

Supplementary Figure 5

Knock-in Rates (Gels, Gel Plot Lines and Band Intensity Analysis)

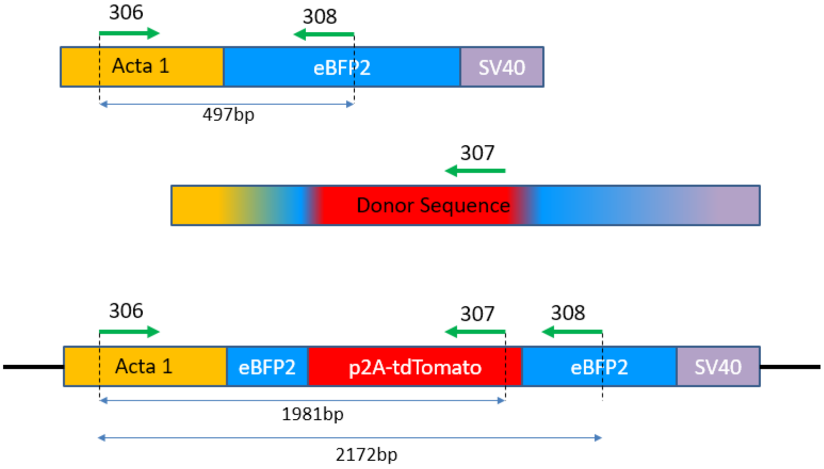

sgRNA		+	+	+	+	+	+	+	+	-	-	-	-	
Cas9		+	+	+	+	+	+	+	+	+	+	+	+	
tdTomato		+	+	+	+	+	+	+	+	+	+	+	+	
Nu7441		+	+	+	+	-	-	-	-	-	-	-	-	

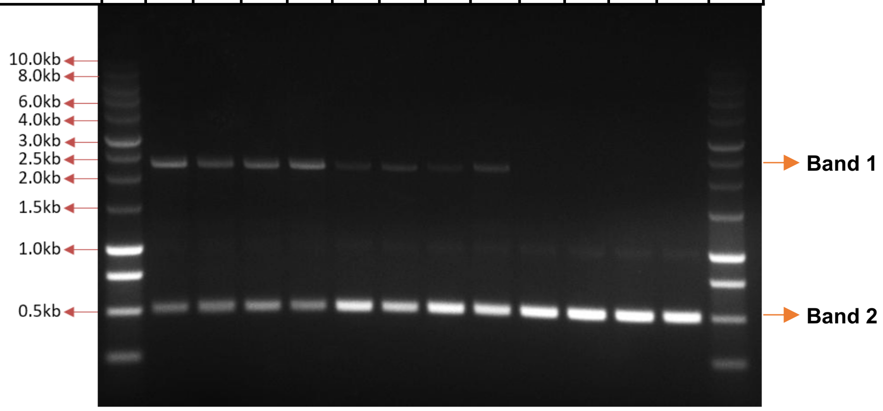

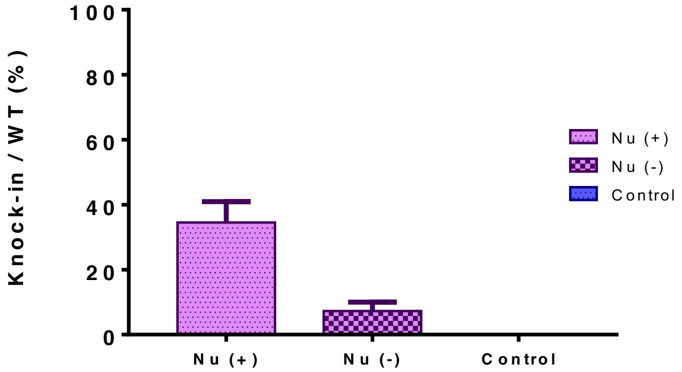

In this experiment, we showed the relative efficiency of knock-in rates tdTomato expressing fast muscle fibers in Nu7441-injected embryos compared to embryos injected without Nu7441 and control group.

Gel:

1) Lane# 1-4: Nu (+) (WT: Crispr/Cas9 + tdTomato + Nu7441)
2) Lane# 5-8: Nu (-) (WT: Crispr/Cas9 + tdTomato)
3) Lane# 9-12: Control (WT: Cas9 + tdTomato)

Supplementary Figure 6

Uncropped gels.

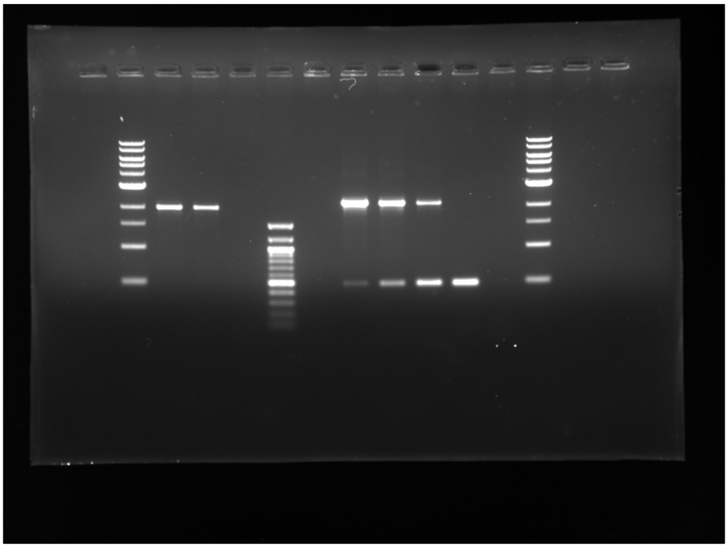

Figure 4D and 4E

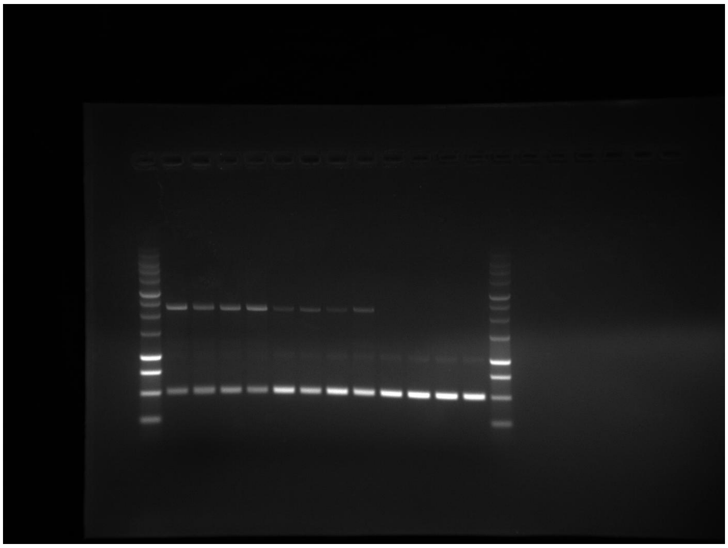

Supplementary Figure 5

Supplementary Table 1

ID	Sequence	Target	Cutting Efficiency (%)
sg_eBFP2_01	GGCCACAAGTTCAGCGTGAGGGG	eBFP2	n/a
sg_eBFP2_02	CCACCGGCAAGCTGCCCGTGCCC	eBFP2, eGFP	37.80
sg_eBFP2_03	CCACGGCGTGCAGTGCTTCGCCC	eBFP2	12.17
sg_eBFP2_04	CCGCTACCCCGACCACATGAAGC	eBFP2, eGFP	56.18
sg_eBFP2_05	CCGCCATGCCCGAAGGCTACGTC	eBFP2, eGFP	6.86
sg_eBFP2_06	CCCCATCGGCGACGGCCCCGTGC	eBFP2, eGFP	26.33

Cutting efficiency of six sgRNAs targeting different loci on eBFP2 gene was determined using RFLP and further validated by observing the loss of blue fluorescence signal from fast-muscle fibers.

Supplementary Table 2

Germline Transmission Rates

Groups	(+)	Total	Germline Transmission Rate (%)	Chi-square p-value (vs -/-)
**Control**	0	8	**0 %**	
**Nu / RS1(-/-)**	1	16	**6.25 %**	
**Nu / RS1(+/-)**	3	6	**50.0%**	**0.018**
**Nu / RS1 (+/+)**	4	10	**40.0%**	**0.034**

Supplementary Table 3

Primers	5’ Sequence 3’	
sg_eBFP2_01_Fwd	TAGGGGCCACAAGTTCAGCGTGAG	gRNA Oligo
sg_eBFP2_01_Rev	AAACCTCACGCTGAACTTGTGGCC	gRNA Oligo
sg_eBFP2_02_Fwd	TAGGGGGCACGGGCAGCTTGCCGG	gRNA Oligo
sg_eBFP2_02_Rev	AAACCCGGCAAGCTGCCCGTGCCC	gRNA Oligo
sg_eBFP2_03_Fwd	TAGGGGGCGAAGCACTGCACGCCG	gRNA Oligo
sg_eBFP2_03_Rev	AAACCGGCGTGCAGTGCTTCGCCC	gRNA Oligo
sg_eBFP2_04_Fwd	TAGGGCTTCATGTGGTCGGGGTAG	gRNA Oligo
sg_eBFP2_04_Rev	AAACCTACCCCGACCACATGAAGC	gRNA Oligo
sg_eBFP2_05_Fwd	TAGGGACGTAGCCTTCGGGCATGG	gRNA Oligo
sg_eBFP2_05_Rev	AAACCCATGCCCGAAGGCTACGTC	gRNA Oligo
sg_eBFP2_06_Fwd	TAGGGCACGGGGCCGTCGCCGATG	gRNA Oligo
sg_eBFP2_06_Rev	AAACCATCGGCGACGGCCCCGTGC	gRNA Oligo
306	GAACACTGCTCTTTCAGATGCCAC	PCR
307	GTCCATGCCGTACAGGAACA	PCR
308	ACTTGAAGAAGTCGTGCTGCT	PCR
270 Seq F	AGGATGTGGCGTGTGCATTA	Sequencing
274 Seq_R	GCTTGCTGAAGGTAGGGGGT	Sequencing
eGFP_SRV1_F	CCCATCCTGGTCGAGCTGGAC	RFLP
eGFP_SRV1_R	AAGAAGATGGTGCGCTCCTGGA	RFLP
eGFP_SRV2_F	TGGTGCCCATCCTGGTCGAG	RFLP
eGFP_SRV2_R	GCCGTCGTCCTTGAAGAAGA	RFLP
eGFP_HRM1_F	CGACGTAAACGGCCACAAGTT	HRM
eGFP_HRM1_R	CCGGTGGTGCAGATGAACT	HRM
eGFP_HRM2_F	CACAAGTTCAGCGTGTCCGGC	HRM
eGFP_HRM2_R	CAGGGTGGTCACGAGGGTGG	HRM
