# Supplementary material for: Chemical reprogramming enhances homology-directed genome editing in zebrafish embryos
Source: Commun Biol. 2019 May 23;2:198. doi: 10.1038/s42003-019-0444-0 (PMC6533270; doi:10.1038/s42003-019-0444-0)
Supplement: Supplementary file 2 — Description of additional supplementary items [file 42003_2019_444_MOESM2_ESM.docx]

Supplementary Movie Legends

**Supplementary Movie 1**

Transgenic *acta1:eBFP2* embryo injected with an eBFP2-targeting sgRNA and Cas9 shows loss of eBFP2 expression in fast-muscle fibers.

**Supplementary Movie 2**

Transgenic *acta1:eBFP2;smyhc1:eGFP* embryo co-injected with eBFP2 sgRNA, Cas9 and DNA donor template exhibits tdTomato expression in individual fast muscle fibers.

**Supplementary Movie 3**

3D-rendered z-stack of a control *acta1:eBFP2;smyhc1:eGFP* embryo injected with Cas9 and DNA donor template but lacking sgRNA.

**Supplementary Movie 4**

Confocal cross-section through an *acta1:eBFP2;smyhc1:eGFP* embryo showing mutually exclusive eBFP2 and eGFP expression in individual fast-muscle fibers.

**Supplementary Movie 5**

3D-rendered z-stack images of transgenic embryos show loss of eGFP and eBFP2 signals at slow- and fast-muscle fibers, respectively. Seamless integration of tdTomato was only observed in fast-muscle fibers.

**Supplementary Data 1**

Complete dataset showing the individual data points that were plotted in the main and supplementary figures.
